# Acceptability and Feasibility of Geographically Explicit Ecological Momentary Assessment Among Men Who Have Sex with Men

**DOI:** 10.1007/s10508-021-02159-6

**Published:** 2021-11-15

**Authors:** Isabelle Sheck, Carla Tilchin, Jessica Wagner, David H. Epstein, Albert Burgess-Hull, Jacky M. Jennings

**Affiliations:** 1 Center for Child and Community Health Research (CCHR), Department of Pediatrics, Johns Hopkins School of Medicine, The Johns Hopkins University Bayview Medical Center, 5200 Eastern Avenue, Mason F. Lord Bldg, Suite 4200, Baltimore, MD 21224 USA; 2 Department of Health, Behavior and Society, Johns Hopkins Bloomberg School of Public Health, Baltimore, MD USA; 3 Intramural Research Program, National Institute on Drug Abuse, The Johns Hopkins University Bayview Medical Center, Baltimore, MD USA; 4 Department of Epidemiology, Johns Hopkins Bloomberg School of Public Health, Baltimore, MD USA

**Keywords:** mHealth, Men who have sex with men (MSM), Geographically explicit ecological momentary assessment (GEMA), Syphilis, HIV

## Abstract

Syphilis among men who have sex with men (MSM) has increased greatly in the past twenty years in the U.S. Geographically explicit ecological momentary assessment (GEMA), in which behaviors are geotagged and contextualized in time and space, may contribute to a greater understanding of transmission risk. The objective was to determine the acceptability and feasibility of GEMA for assessing HIV and syphilis transmission risk behaviors among a sample of MSM. Participants responded to a brief survey five times a day for two weeks. Feasibility was measured by participant recruitment, enrollment, prompts received and answered, geotagged prompts, and technical interference with data collection. Acceptability was measured by ratings of enjoyment and willingness for future participation. Summaries of five behavioral measures from the brief survey were calculated. Among the 83 participants contacted, 67.5% (56) expressed interest, 98% (55) were scheduled, and 81.8% (45) were enrolled. Participants answered 78.3% (2,277) of prompts received and 87.7% (1,998) of answered prompts were geotagged. Overall, 70.5% (31) enjoyed participating and 91.1% (41) were willing to participate in the future. Among prompts answered, missingness was low for five behavioral measures (range 0.2% (4) to 0.7% (16)). Feasibility and acceptability were high and missingness was low on behavioral measures in this MSM study population. Most participants reported that they would participate again. Future work should focus on whether GEMA improves our understanding of syphilis and HIV transmission risk.

## Introduction

Syphilis among gay, bisexual and other men who have sex with men (MSM) has increased greatly in the United States (U.S.) over the past twenty years (Centers for Disease Control and Prevention [CDC], 2019). Primary and secondary syphilis rates have increased almost every year since 2001, and in 2019, 56.7% of cases were among MSM (CDC, 2020, 2021). Reported cases of syphilis continue to be characterized by a high rate of HIV co-infection, particularly among MSM (CDC, 2019). While data suggest that multiple factors may be contributing to the syphilis and HIV co-infection epidemic among MSM, a handful of studies suggest that drug use and specifically stimulant use, such as methamphetamine use, in combination with sex may be a significant contributing factor (Drückler et al., 2018; Landovitz et al., 2013; Bourne et al., 2018; Race, 2015; Chew et al., 2013; Klitzman et al., 2000; Stall & Purcell, 2000; Waldo et al., 2000; Jennings et al., 2021).

One limitation of current prevention and control efforts may be related to the data collection methods utilized to understand behaviors such as substance use. For example, standard data collection techniques rely on retrospective recall periods of three-, six- or twelve-month time periods. The retrospective nature and length of recall time may lead to recall bias and be mismatched to sexual behavior, substance use, and other transmission risk behaviors which may vary more frequently, such as on an hourly, daily, or weekly basis (Wray et al., 2016). In addition, standard techniques may be limited in their ability to collect accurate data on behaviors, which may be influenced by context.

Real-time assessment tools defined as intensive longitudinal methods (ILM), including daily diary, event-based and ecological momentary assessments (EMA), may represent a significant improvement in the measurement of behaviors related to transmission risk. Traditional measurement methods are often limited by single measurements in time or few repeated measurements were taken over long-time intervals, whereas, ILM measurements increase the frequency of measurements often in a short period of time allowing for the measurement of thoughts, feelings, and behavior in their natural, spontaneous contexts (Bolger & Laurenceau, 2013; Fraley & Hudson, 2014). Geographically explicit EMA (GEMA) is one type of ILM data collection method that ‘accompanies’ participants by capturing geolocation data and providing frequent survey prompts as a participant moves through time and space. GEMA may be useful to measure behaviors in real-time that are likely fluid, dynamic, and vary by context (Duncan et al., 2019; McQuoid et al., 2019; Shiffman et al., 2008; Wray, 2016). For example, the use of GEMA may lend insight to variability in sexual and substance use behaviors and the motivations behind the behaviors, as well as how these behaviors may vary within the context of specific relationships (e.g. casual, anonymous, main) and specific settings (i.e. sex partner meeting places) (Jones, 2019; Turner et al., 2017; Alabduljader et al., 2018; Richard et al., 2017).

While there have been studies suggesting high acceptability and feasibility of similar methods to GEMA, these studies did not assess the feasibility of capturing geographic information to inform behavior in context (Turner et al., 2017; Hubach et al., 2020; Wray, 2016). Given the high burden of infection, the complexity of assessment of behaviors in time and space, and the limitations in prior studies to-date, the objective was to determine the acceptability and feasibility of GEMA for assessing HIV and syphilis transmission risk behaviors among a sample of MSM. The overall goal of this research is to improve the assessment of dynamic factors and contexts contributing to HIV and STI transmission risk behaviors among MSM.

## Methods

### Overview

This pilot study was nested within a parent study, the Understanding Sexual Health in Networks (USHINE) study, which is an ongoing longitudinal cohort of MSM designed to inform the network epidemiology of syphilis transmission. The parent study and pilot were approved by the Johns Hopkins School of Medicine IRB.

Participants in the parent study were recruited from three clinical sites, including one primary care site and two public sexual health clinics, and one community-based organization in one mid-Atlantic city, Baltimore City, Maryland. Individuals were eligible to participate if they reported male sex at birth, current male gender, age 18–45, sex with a man in the past six months, residence in Baltimore City, and were willing and able to give informed consent for the study. The parent study involves a baseline visit and quarterly follow-up visits for up to two years and includes a survey as well as biological testing for syphilis and HIV at each study visit. Syphilis positivity for this study was defined as a rapid plasma reagin (RPR) titer greater than 1:8. HIV positivity was defined as a positive HIV rapid test with ELISA confirmation and/or medical record documentation of a prior positive HIV diagnosis at any study visit.

The pilot study was conducted from January 15, 2020 to March 23, 2020. Pilot study participants were eligible for the pilot if they consented for recontact for additional research at enrollment, reported going to at least one sex partner meeting place (i.e., venue), and had documentation of HIV and syphilis status. From this pool of eligibles, potential participants were randomly selected for participation from four groups based on syphilis and HIV infection status in the parent study (i.e., syphilis positive and HIV negative, syphilis positive and HIV positive, syphilis negative and HIV positive, syphilis negative and HIV negative).

The selection process ensured an adequate sample in each group, such as in the syphilis positive and HIV-negative group, in order to allow for future exploratory analyzes comparing these groups.

### Data Collection

Data were collected through two main methods. The first was a GEMA application which included programed prompts to respond to a brief survey (Epstein et al., 2009, 2014). The second was an exit survey developed using REDCap (Research electronic data capture), an electronic data capture tool to support clinical and translational research (Harris et al., 2009). The brief survey included 16–32 questions depending on which behaviors were reported, and the exit survey included 22 questions.

### Procedures

After providing informed consent and agreeing to participate in the pilot study, participants were asked for their waking hours to allow for the programming of prompts five times a day (every 3–4 h) for 14 days within self-reported waking hours. Each participant was then given a Samsung A20 Galaxy cell phone, and participants were prompted to respond to each brief survey. Participants were informed that each assessment would take five to ten minutes. To minimize technical issues experienced during the study, research assistants conducted a brief training session including a practice trial on the mobile device and general tips for successful completion of the protocol. Participants were encouraged to text or call research staff if they encountered any issues or had any questions. Participants received remuneration for completing at least 80% of GEMA prompts received each week during the two-week protocol and for returning the mobile device upon study completion. Participants were informed at enrollment that failure to comply with the study protocol could impact their payment.

### Measures

Measures to describe study participants were obtained from the parent study baseline survey including age, race, ethnicity, education, employment, and sexual and substance risk behaviors in the past three months.

Self-reported measures adapted from existing measures were utilized to assess GEMA acceptability and feasibility (Burke et al., 2017; Duncan et al., 2019; Hubach et al., 2020; Yang et al., 2015), and data are presented for feasibility overall and by week to assess whether interest waned over time. Feasibility was measured by participant recruitment and enrollment and the number and proportion of received, answered, and geotagged prompts overall and by week. Prompts were not received if the participant’s phone was not on, the participant did not have service, or if there were other technical issues with the GEMA application. Received prompts were automatically logged by the GEMA application. Geolocation data included latitude, longitude, and altitude and was captured every fifteen minutes and at every brief survey prompt. Feasibility measures also included the number and proportion of participants who experienced technical issues and the degree to which technical issues impacted data collection.

Acceptability was measured by self-reports of study enjoyment and willingness for future participation, in an exit survey provided to participants when they returned their phones. Acceptability was also measured by whether participants thought the surveys were too long, random prompts were an interruption to daily activities and/or too frequent, carrying the phone was too burdensome, and whether they were interested in participating again in the future. Exit survey questions measuring acceptability and feasibility were asked with a 5-point Likert scale ranging from Strongly Agree to Strongly Disagree, except for one Yes/No question and a corresponding free text option to describe any technical issues experienced during the 2-week protocol.

Because feasibility and acceptability may have been impacted by the type of questions and length of the brief survey, and ultimately, the goal is to capture real-time behaviors in context, five selected behavioral measures from the brief survey are provided on sexual behavior, substance use, mood and emotion, activities since last entry and use of online apps. Each measured behaviors since last entry and offered multiple-choice response options including “other” followed by a free text response option. The multiple-choice response options were informed by the most frequent responses to similar questions on the parent survey and were allowed participants to “Check all that apply” when applicable. In addition, at the end of the exit survey, one free text response option was included, “Please let us know if there is any other information about your experience that you would like to share with us.”

### Statistical Testing

Basic characteristics of enrolled versus not enrolled participants were compared using chi-square and Mood’s median test for continuous variables with a non-normal distribution (Mood, 1954). Summary statistics were generated to measure feasibility overall and by each week, acceptability, and the five brief survey measures. Analyzes were performed using Stata version 15 (Stata Corp., College Station, Texas) and statistical significance was defined as a *p*-value < .05. We did not have hypotheses regarding differences by infection group, and therefore, we did not conduct statistical testing by the group.

## Results

### Study Population

Among USHINE participants, 279 were eligible for the pilot study including 6.1% (17) who were syphilis positive and HIV negative, 10.3% (29) who were syphilis positive and HIV positive, 29.7% (83) who were syphilis negative and HIV positive, and 53.9% (150) who were syphilis negative and HIV negative. Within each infection group, approximately 20 participants were randomly selected to participate with the goal of ultimately enrolling approximately ten to eleven participants in each group. Eighty-three participants were contacted to participate in the pilot and among these, 67.5% (56) expressed interest, 32.5% (27) did not respond to study staff, and 5.4% (3) declined to participate, citing the burden of the study during work hours or insufficient payment. Among the 56 who expressed interest, 98% (55) were scheduled, of whom 81.8% (45) were enrolled. The median number of contact attempts made by study staff to schedule a participant enrollment visit was one (IQR:1). Individuals who were contacted but did not enroll did not differ significantly by characteristics included in Table 1 from those who did enroll.

**Table 1:** Characteristics of participants enrolled (*N* = 45) in the two-week GEMA pilot study, Baltimore City, January 21, 2020 to March 23, 2020

Characteristics	Enrolled *N* = 45
*Demographics*	
Age, mean (SD)	29.7 (5.59)
	N (%)
Race, Black	39 (86.7)
Ethnicity, Latinx	2 (4.4)
Education, > high school education	21(46.7)
Employment, full-time	21(46.7)
*Sexual and substance risk behaviors, past 3 months*	
Number of sex partners, median (IQR)	3 (3)
	N (%)
Condom use, last sex	19 (51.3)
Number of receptive anal sex acts, median (IQR)	5 (12)
Non-injection drug use^a^	12 (26.7)
Injection drug use^b^	3 (6.7)
*Syphilis/HIV infection status*^*c*^	
Syphilis positive HIV positive	12 (26.7)
Syphilis positive, HIV negative	12 (26.7)
Syphilis negative, HIV positive	10 (22.2)
Syphilis negative, HIV negative	11 (24.4)

^a^ Non-injection drug use defined as self-reported consumption (sniff, snort, and inhale) of heroin, cocaine, methamphetamines, marijuana, and/or other drugs not prescribed by a health care provider, within the last three months

^b^ Injection drug use defined as self-reported injection of heroin, cocaine, methamphetamines, and/or other drugs not prescribed by a health care provider, within the last three months

^c^ Syphilis positivity was defined as a rapid plasma reagin (RPR) titer greater than 1:8 and HIV positivity was defined as a positive HIV rapid test with ELISA confirmation and/or medical record documentation of a prior positive HIV diagnosis at any study visit

Among the 45 enrolled, the mean age was 29.7 (SD: 5.59), 86.7% (39) were Black and 4.4% (2) were Latinx (Table 1). Nearly 47% (21) had above a high school education and 46.7% (21) were employed full-time. In the past three months, the median number of sex partners was 3 (IQR: 3), and the median number of receptive anal sex acts was 5 (IQR: 12.0). Fifty-one percent (19) of participants reported using a condom at last sex. A total of 26.7% (12) reported non-injection drug use and 6.7% (3) reported injection drug use in the past three months. As a result of the sampling from infection groups, 26.7% (12) were syphilis positive and HIV positive, 26.7% (12) were syphilis positive and HIV negative, 22.2% (10) were syphilis negative and HIV positive, and 24.4% (11) were syphilis negative and HIV negative.

### Feasibility Assessment

A total of 45 participants were enrolled for the pilot study and 91.1% (41) completed the full protocol. Nine percent (4) were unable to complete the full protocol including one participant who reported unforeseen personal circumstances, one who was overburdened by technical issues with the phones and survey responses, and two who reported that their phone was stolen.

Assuming all 45 participants would complete the full protocol over the two-week pilot study, 3,150 (1,575 prompts each week) were scheduled to be sent to participant mobile devices (Table 2). In total, 92.3% of prompts (2,909) were successfully received. More prompts were received during week one 99.5% (1568) compared to week two 85.1% (1341) (*p*-value < .001). Participants answered 78.3% (2,277) of prompts received overall. A similar proportion of prompts were answered in week one 77.2% (1,210) and in week two 79.6% (1,067) (*p*-value = .118). Among the 2,277 answered prompts, 87.7% (1,998) had associated GPS data. The same percentage of prompts had associated GPS data during week one compared to week two [87.7% (1061), 87.8% (937), respectively, *p*-value = .925] (Table 2). From the geotagged data that was captured every 15 min, an additional 44,829 GPS points were captured outside of prompts.

**Table 2:** Feasibility measured by the number and percentage^a^ of received, answered, and geotagged prompts^b^ by participants (*N* = 45) overall and by week in the two-week GEMA pilot study, Baltimore City, January 21, 2020 to March 23, 2020

	Scheduled prompts N	Prompts received N (%)	Prompts answered N (%)	Geotagged Prompts** N (%)
Overall	3150	2909 (92.3)	2277 (78.3)	1998 (87.7)
Week 1	1575	**1568 (99.5)**	1210 (77.2)	1061 (87.7)
Week 2	1575	**1341 (85.1)**	1067 (79.6)	937 (87.8)

Bold indicates *p*-value < .05

^a^ Percentages defined as the count within the column over the preceding column denominator

^b^ Geotagged prompts defined as prompts that were answered by a participant and had a GPS data point associated with the prompt

Fifty-six percent (25) of participants indicated on their exit survey that they experienced technical difficulties and 84.0% (21) reported the issue to the research assistant during the two-week pilot (Table 3). The most common issue reported by 16.7% (7) participants was being unable to open the survey on the mobile device when they were prompted. Additionally, 9.5% (4) reported not receiving scheduled survey prompts, and 7.1% (3) reported that mobile device prompt notifications (ringing, vibrating) continued during and after the survey was already completed. These issues were documented by study staff and staff worked with participants to remedy the issues. Despite technical difficulties, participants reporting vs. not reporting technical issues received a similar proportion of prompts (91.7% (1605) vs. 93.1% (1304), *p* = .89) and answered a similar proportion of prompts (75.6% (1220) vs. 81.1% (1057), *p* = .24), respectively. Phones were returned after a median of one day (IQR = 4) after data collection was complete.

**Table 3:** Self-reported participant feasibility and acceptability responses in the exit survey upon completion of the two-week GEMA pilot study, Baltimore City, January 21, 2020 to March 23, 2020 (*N* = 45)

	N (%)	N (%)	N (%)
*Feasibility*	Yes	No	Refused to Answer
Experienced technical issues	25 (59.5)	16 (38.1)	1 (2.4)
*Acceptability*	Agree	Neutral	Disagree
Enjoyed study participation	31 (70.5)	6 (13.6)	7 (15.9)
Among those that experienced technical issues (n = 25), enjoyed study participation despite technical difficulties	19 (76.0)	4 (16.0)	2 (8.0)
Questionnaires were too long	3 (6.7)	12 (26.7)	30 (66.7)
Random prompts were an interruption in my daily activities	17 (37.8)	10 (22.2)	18 (40.0)
Random prompts were too frequent	13 (28.9)	10 (22.2)	22 (48.9)
Carrying the phone was burdensome for me	9 (20.0)	7 (15.6)	29 (64.4)
	Yes	No	Refused to Answer
Would you like to participate again in the future?	41 (91.1)	1 (2.2)	3 (6.7)

### Acceptability Assessment

One hundred percent (45) of participants completed the exit survey. Seventy-one percent (31) of participants enjoyed participating in the study (Table 3). Among those experiencing technical issues (25), 76.0% (19) enjoyed study participation despite the technical difficulties. Overall, 66.7% (30) disagreed with the statement that the “Questionnaires were too long.” Forty percent (18) disagreed with the statement that the “Random prompts were an interruption to my daily activities,” and 48.9% (22) disagreed with the statement that the “Random prompts were too frequent.” Sixty-four percent (29) disagreed with the statement that “Carrying the phone was burdensome for me,” and 91.1% (41) wanted to participate again in the future. Twenty-two percent (10) of participants provided free text responses in the exit survey. Five participants expressed that the study was interesting, informative, a good experience, and/or that they would enjoy participating again, including one who also reported that the questions helped them reflect on their use of online sex partner meeting venues and drugs that they used on a weekly basis. Two participants reported frustration with receiving erroneous brief survey prompt notifications during and immediately after completing the survey, and another suggested adding a “snooze” button to silence notifications sent during driving or in transit. One participant expressed their preference to have the application downloaded on their own mobile device (vs. the study mobile device). Another participant felt the survey questions needed to be modified to capture more useful information and another felt that the results may not reflect “normal” circumstances since the study only took place over a two-week period.

Among prompts received and answered (2277), missingness across the five selected behavioral measures was low including 0.2% (4) for activity, 0.6% (13) for sexual behavior, 0.7% (16) for substance use, 0.4% (10) for mood/emotion, and 0.6% (13) for online app use. Since last entry (i.e., three to four hours prior), participants reported being intimate or having sex in 2.9% (65) of prompts and having had oral sex in 3.8% (86) of prompts (Table 4). For substance use, participants reported marijuana use in 18.7% (422) of prompts and methamphetamine use in 2.0% (46) of prompts. Participants reported being horny, aroused and/or excited in 6.6% (150) of prompts and intoxicated, drunk and/or high in 4.5% (102) of prompts. The most frequently reported online app usage since last entry included Facebook [25.6% (579)], Instagram [19.1% (432)], and Grindr [11.8% (268)] (Table 4).

**Table 4:** Selected participant behaviors reported since last prompt (i.e. three to four hours) during the two-week GEMA pilot study, Baltimore City, January 21, 2020 to March 23, 2020 (participant *N* = 45, prompts *N* = 2,277)

Behaviors since last prompt (i.e. 3–4 h prior)	*N*	**%**
*Sexual Behavior (N* = *2411)*		
Masturbated	196	8.7
Oral	86	3.8
Anal	69	3.1
Sexted	58	2.6
Some other type of sex^a^	30	1.3
None of the above	1963	86.7
*Substance Use (N* = *2420)*		
Marijuana	422	18.7
Alcohol	131	5.8
Methamphetamines	46	2.0
Prescription pain killers	45	2.0
Other^b^	31	1.4
I haven’t used any drugs	1707	75.5
*Mood and Emotion (N* = *2955)*		
Relaxed/content/at ease	1107	48.8
Hungry/tired/worn out	799	35.2
Horny/aroused/excited	150	6.6
Angry/anxious/upset	142	6.3
Intoxicated/drunk/high	102	4.5
Other	655	28.9
*Activities (N* = *2769)*		
Working	655	28.8
Socializing	483	21.3
Talking online/browsing social media	369	16.2
Making plans to go out	116	5.1
Being intimate or having sex	65	2.9
Other	1081	47.6
*Sex partner meeting venues (N* = *3058)*		
Facebook	579	25.6
Instagram	432	19.1
Grindr	268	11.8
Jack’d	109	4.8
Tagged	107	4.7
Adam4Adam	92	4.1
Other^c^	143	6.3
I haven’t been online	1267	56.0

All questions were “Check all that apply”, creating total numbers of behavioral responses larger than the total numbers of prompts

^a^ Some other type of sex defined any item with < 2% in responses including group sex and vaginal sex and other

^b^ Other defined any item with < 2% in responses including prescription erectile dysfunction, ecstasy, PCP, crack/cocaine, heroin, poppers, and other

^c^ Other defined as any item with < 2% in responses including Tinder, Scruff, Tumblr, Kik and other

## Discussion

Syphilis continues to infect MSM at a significantly higher rate than other populations, suggesting a critical need to address this public health crisis with innovative methods (CDC Surveillance Report, 2019). This pilot study sought to determine the feasibility and acceptability of the use of GEMA among MSM. This method allows for intensive data collection which may have the potential to provide detailed information on sexual and drug behaviors in time and space and yield new interventions.

Overall, there was a high willingness to participate in a GEMA among this study population of urban and predominantly Black MSM. Participants answered the majority of prompts received and the majority of answered prompts were geotagged during the two-week period. The proportion of answered prompts (78%) was slightly higher than the response rate to daily surveys (74%) shown in previous studies of EMA among MSM (Turner et al., 2017; Yang et al., 2015). The overwhelming majority of participants said that they would participate again in the future and most indicated that they enjoyed participating in the study. While just over half reported technical issues during the study, there were no differences in the proportion of prompts received and answered among those experiencing versus not experiencing technical difficulties. Most participants thought the number of prompts was acceptable and did not find that prompts were an interruption to daily activities. Among the selected measures, the completion rate for each question was high, demonstrating the feasibility and acceptability of this mode of collection of information related to sexual and drug behaviors, mood and emotion, activities since last entry, and use of online apps.

While emerging research has used GPS and EMA methods separately among MSM, less is known about the acceptability and feasibility of combining these methods and specifically about whether participants would be willing to have their survey data accompanied by geotagged information (Duncan et al., 2017, 2019; Hubach et al., 2020; Turner et al., 2017). Studies also suggest that participants may be more likely to share information about socially sensitive behaviors like substance use and sexual behaviors via electronic modalities (i.e., mHealth) like the one utilized in this pilot (Bonar et al., 2018; Duncan et al., 2019) compared to traditional data collection techniques. In other study protocols utilizing mHealth technology interventions in substance use and HIV-related research, participants did not have significant privacy concerns with sharing sensitive information and reported benefits from mHealth interventions including convenience, comfort discussing sensitive topics, and privacy (Bonar et al., 2018).

GEMA may have a greater potential to evaluate highly intensive behaviors, like online application use among MSM. Real-time geographic location tracking with corresponding EMA data provides an opportunity to capture nuanced spatial–temporal links between behavior and context among MSM, data that is frequently lost in retrospective assessment (Duncan et al., 2019; Shiffman et al., 2008; Wray, 2016). High acceptability for assessing these behaviors in conjunction with tracking geographic location will continue to be increasingly important as online apps and mHealth prevention and intervention technologies become more widely used.

There were several limitations to this pilot study. First, additional research will need to determine how meaningful variability in sexual and substance use behaviors captured by high-density, real-time assessments compare with more traditional and longer time frame methods. While acceptability was generally high, we did not specifically assess the acceptability of GPS data collection. As part of the enrollment process, we made sure to explain to participants that location and other information would be collected and utilized solely for the purposes of this study. Although the current study enrolled more participants than many existing EMA and GEMA protocols with MSM, future work including larger sample sizes is needed to increase the generalizability of these results. Lastly, just under one third of participants contacted for the pilot did not respond. Many of the non-respondents, however, had already missed several parent study visits, suggesting they may have been lost to follow-up in the parent study instead of explicitly disinterested in participation in the pilot study, and those that were enrolled were not different on basic characteristics than those not enrolled.

Despite these limitations, participant enjoyment and engagement in the study demonstrated that this method is acceptable and feasible among MSM and may be an important tool to assess sexual and drug behavior in time and space. Given the overall high approval of the protocol and low missingness of responses to behavioral questions, GEMA presents an alternative to traditional retrospective assessment methods and may provide context to decisions that lead up to, take place during, and occur after a sexual encounter. This type of information is crucial for designing effective disease prevention and transmission interventions, especially for populations experiencing high rates of diseases such as syphilis and HIV among MSM. Future investigations should build on the current findings of acceptability and feasibility to develop just-in-time adaptive interventions (JITAI) that aim to provide individualized support to MSM and other underserved populations (Chaix, 2020; Nahum-Shani et al., 2018).

## Data Availability

With the appropriate approvals by the IRB and institutional Data Trust, a de-identified set of data may be made available.

## Code Availability

Available upon request.

## References

[CR1] Alabduljader K, Cliffe M, Sartor F, Papini G, Cox W, Kubis H (2018). Ecological momentary assessment of food perceptions and eating behavior using a novel phone application in adults with or without obesity. Eating Behaviors.

[CR2] Bolger N, Laurenceau J (2013). Intensive longitudinal methods: An introduction to diary and experience sampling research.

[CR3] Bonar E, Koocher G, Benoit M, Collins R, Cranford J, Walton M (2018). Perceived risks and benefits in a text message study of substance abuse and sexual behavior. Ethics and Behavior.

[CR4] Bourne A, Ong J, Pakianathan M (2018). Sharing solutions for a reasoned and evidence-based response: Chemsex/party and play among gay and bisexual men. Sexual Health.

[CR5] Burke L, Shiffman S, Music E, Styn M, Kriska A, Smailagic A (2017). Ecological momentary assessment in behavioral research: addressing technological and human participant challenges. Journal of Medical Internet Research.

[CR6] Centers for Disease Control and Prevention. (2019). Sexually transmitted disease surveillance 2018. Atlanta: U.S. Department of Health and Human Services. doi: 10.15620/cdc.79370.

[CR7] Centers for Disease Control and Prevention. (2021). Sexually Transmitted disease surveillance 2019. Atlanta: U.S. Department of health and human services. https://www.cdc.gov/std/statistics/2019/overview.htm

[CR8] Centers for Disease Control and Prevention. (2020). Estimated HIV incidence and prevalence in the United States, 2014–2018*.* HIV Surveillance Supplemental Report, *25*(1). http://www.cdc.gov/ hiv/library/reports/hiv-surveillance.html.

[CR9] Chaix B (2020). How daily environments and situations shape behaviors and health: Momentary studies of mobile sensing and smartphone survey data. Health & Place.

[CR10] Chew Ng RA, Samuel M, Lo T, Bernstein K, Anyalem G, Klausner J (1971). Bolan G (2013) Sex, drugs (methamphetamines), and the internet: Increasing syphilis among men who have sex with men in California 2004-2008. American Journal of Public Health (1971).

[CR11] Drückler S, van Rooijen M, de Vries H (2018). Chemsex among men who have sex with men: A sexualized drug use survey among clients of the sexually transmitted infection outpatient clinic and users of a gay dating app in Amsterdam The Netherlands. Sexually Transmitted Diseases.

[CR12] Duncan D, Kapadia F, Kirchner T, Goedel W, Brady W, Halkitis P (2017). Acceptability of ecological momentary assessment among young men who have sex with men. Journal of LGBT Youth.

[CR13] Duncan D, Park S, Goedel W, Sheehan D, Regan S, Chaix B (2019). Acceptability of smartphone applications for global positioning system (GPS) and ecological momentary assessment (EMA) research among sexual minority men. PLOS ONE.

[CR14] Epstein D, Tyburski M, Craig I, Phillips K, Jobes M, Vahabzadeh M, Mezghanni M, Lin JL, Furr-Holden CD, Preston K (2014). Real-time tracking of neighborhood surroundings and mood in urban drug misusers: Application of a new method to study behavior in its geographical context. Drug and Alcohol Dependence.

[CR15] Epstein DH, Willner-Reid J, Vahabzadeh M, Mezghanni M, Lin J, Preston KL (2009). Real-time electronic diary reports of cue exposure and mood in the hours before cocaine and heroin craving and use. Archives of General Psychiatry.

[CR16] Fraley R, Hudson N (2014). Review of intensive longitudinal methods: An introduction to diary and experience sampling research. The Journal of Social Psychology.

[CR17] Harris PA, Taylor R, Thielke R, Payne J, Gonzalez N, Conde JG (2009). Research electronic data capture (REDCap)—a metadata-driven methodology and workflow process for providing translational research informatics support. Journal of Biomedical Informatics.

[CR18] Hubach R, O’Neil A, Stowe M, Giano Z, Curtis B, Fisher C (2020). Perceived confidentiality risks of mobile technology-based ecologic momentary assessment to assess high-risk behaviors among rural men who have sex with men. Archives of Sexual Behavior.

[CR19] Jennings JM, Wagner J, Tilchin C, Schumacher CM, Thornton N, Hamill M, Rompalo A, Ruhs S, Rives S, Ghanem KG, Latkin C (2021). Methamphetamine use, syphilis and specific online sex partner meeting venues are associated with HIV status among urban black gay and bisexual men who have sex men. Sexually Transmitted Diseases.

[CR20] Jones MLJ (2019). Extragenital chlamydia and gonorrhea among community venue-attending men who have sex with men—five cities, United States, 2017. MMWR. Morbidity and Mortality Weekly Report.

[CR21] Klitzman RL, Pope HG, Hudson JI (2000). MDMA ("ecstasy") abuse and high-risk sexual behaviors among 169 gay and bisexual men. The American Journal of Psychiatry.

[CR22] Landovitz R, Tseng C, Weissman M, Haymer M, Mendenhall B, Rogers K, Shoptaw S (2013). Epidemiology, sexual risk behavior, and HIV prevention practices of men who have sex with men using GRINDR in Los Angeles California. Journal of Urban Health.

[CR23] McQuoid J, Thrul J, Ozer E, Ramo D, Ling P (2019). Tobacco use in the sexual borderlands: The smoking contexts and practices of bisexual young adults. Health and Place.

[CR24] Mood A (1954). On the asymptotic efficiency of certain nonparametric two-sample tests. The Annals of Mathematical Statistics.

[CR25] Nahum-Shani I, Smith SN, Spring BJ, Collins LM, Witkiewitz K, Tewari A (2018). Just-in-time adaptive interventions (JITAIs) in mobile health: Key components and design principles for ongoing health behavior support. Annals of Behavioral Medicine.

[CR26] Race K (2015). ‘Party and Play’: Online hook-up devices and the emergence of PNP practices among gay men. Sexualities.

[CR28] Richard A, Meule A, Reichenberger J, Blechert J (2017). Food cravings in everyday life: An EMA study on snack-related thoughts, cravings, and consumption. Appetite.

[CR29] Shiffman S, Stone A, Hufford M (2008). Ecological momentary assessment. Annual Review of Clinical Psychology.

[CR30] Stall R, Purcell D (2000). Intertwining epidemics: A review of research on substance use among men who have sex with men and its connection to the AIDS epidemic. AIDS and Behavior.

[CR31] Turner C, Coffin P, Santos D, Huffaker S, Matheson T, Euren J, DeMartini A, Rowe C, Batki S, Santos G (2017). Race/ethnicity, education, and age are associated with engagement in ecological momentary assessment text messaging among substance-using MSM in San Francisco. Journal of Substance Abuse Treatment.

[CR32] Wald C, McFarland W, Katz M, MacKellar D, Valleroy L (2000). Very young gay and bisexual men are at risk for HIV infection: The San Francisco bay area young men's survey II. Journal of Acquired Immune Deficiency Syndromes (1999).

[CR33] Wray TB, Kahler CW, Monti PM (2016). Using ecological momentary assessment (EMA) to study sex events among very high-risk men who have sex with men (MSM). AIDS and Behavior.

[CR34] Yang C, Linas B, Kirk G, Bollinger R, Chang L, Chander G, Siconolfi D, Braxton S, Rudolph A, Latkin C (2015). Feasibility and acceptability of smartphone-based ecological momentary assessment of alcohol use among African American men who have sex with men in Baltimore. JMIR mHealth and uHealth.

